# Interactive effect of serum uric acid and handgrip strength on all-cause mortality among Japanese community-dwelling people

**DOI:** 10.1016/j.metop.2022.100227

**Published:** 2022-12-29

**Authors:** Ryuichi Kawamoto, Asuka Kikuchi, Daisuke Ninomiya, Teru Kumagi

**Affiliations:** a Department of Community Medicine, Ehime University Graduate School of Medicine, Toon-city, Ehime, 791-0295, Japan; b Department of Internal Medicine, Seiyo Municipal Nomura Hospital, Seiyo-city, Ehime, 797-1212, Japan

**Keywords:** Uric acid, Handgrip strength, Risk factor, All-cause mortality

## Abstract

**Background:**

Uric acid is both a pro-oxidant and an antioxidant. This study examined whether serum uric acid (SUA) is associated with all-cause mortality and cardiovascular biomarkers in members of the general population who had varying levels of handgrip strength (HGS).

**Methods:**

The analysis is based on 1736 participants, of whom 785 were male (69 ± 11 years old) and 951 were female (69 ± 9 years old). We obtained adjusted relative risk estimates for all-cause mortality from the Japanese Basic Resident Registry and used a Cox proportional hazards model (adjusted for possible confounders) to determine the hazard ratios (HR) and 95% confidence intervals (CI).

**Results:**

The results indicated a significant interaction between the effects of SUA levels and HGS on all-cause mortality risk. Among participants with low HGS (<30.0 kg in males, <20.0 kg in females), low SUA levels (<3.5 mg/dL in males, <3.0 mg/dL in females; HR: 2.40; 95% CI: 1.07–5.40) and high SUA levels (≥8.0 mg/dL in males, ≥7.0 mg/dL in females; HR: 3.05; 95% CI: 1.41–6.59) were associated with a significantly higher HR for all-cause mortality than medium SUA levels (3.5–7.9 mg/dL in males, 3.0–6.9 mg/dL in females). Among participants with high HGS (≥30.0 kg in males; ≥20.0 kg in females), there was no difference between the HR for all-cause mortality between the three SUA-category groups.

**Conclusions:**

The association between SUA and the risk of all-cause mortality was U-shaped for this population of community-dwelling adults. This was primarily true for those with low HGS.

## 1 Introduction

Uric acid (UA) is the final oxidation product of the purine metabolism in humans and is catalyzed by xanthine oxidase, an enzyme linked to oxidative stress. Numerous studies have identified hyperuricemia as a critical determinant of systemic inflammation [1], endothelial dysfunction [2], metabolic syndrome [3], cardiovascular disease (CVD) [4], CVD-related death [5,6], and overall mortality [6,7]. Despite researchers showing that serum UA (SUA) levels are associated with various types of CVD in humans, it is thought that UA not only plays an etiologic role in these pathologies, but also acts as a reactive oxygen species scavenger with strong antioxidant properties, which contributes to the elimination of free radicals [8]. According to Nahas et al. [9,10], higher circulating levels of SUA are likely to be associated with greater handgrip strength (HGS) in middle-aged and older persons. However, clinical studies have suggested that high SUA levels are a marker of better prognosis for Parkinson's disease [11], Alzheimer's disease [12], and fractures [13].

Handgrip strength is a fundamental parameter in biomechanical modeling and has found many valuable applications in sports practice, equipment and consumer product design, and ergonomic tool development [14]. It is important for a person to perform forelimb and precision hand functions and is used as one of the main indicators when testing muscle strength [14]. It is used to diagnose both sarcopenia and frailty [[15], [16], [17]]. Studies have suggested that HGS can accurately and consistently predict all-cause mortality in middle-aged and older adults [[18], [19], [20], [21], [22]]. Among various risk factors, studies have suggested that muscle oxidative stress plays an important role in sarcopenia [23], raising the question of how UA, which has both pro- and antioxidant properties, affects sarcopenia. In this study, we hypothesized that the relationship between SUA levels and the risk of all-cause mortality varies across the spectrum of HGS.

To test this hypothesis, we examined whether there is an association between SUA levels and all-cause mortality, particularly in people with low HGS, a useful indicator of sarcopenia.

## 2 Materials and methods

### 2.1 Study design and participants

**Fig. 1:**
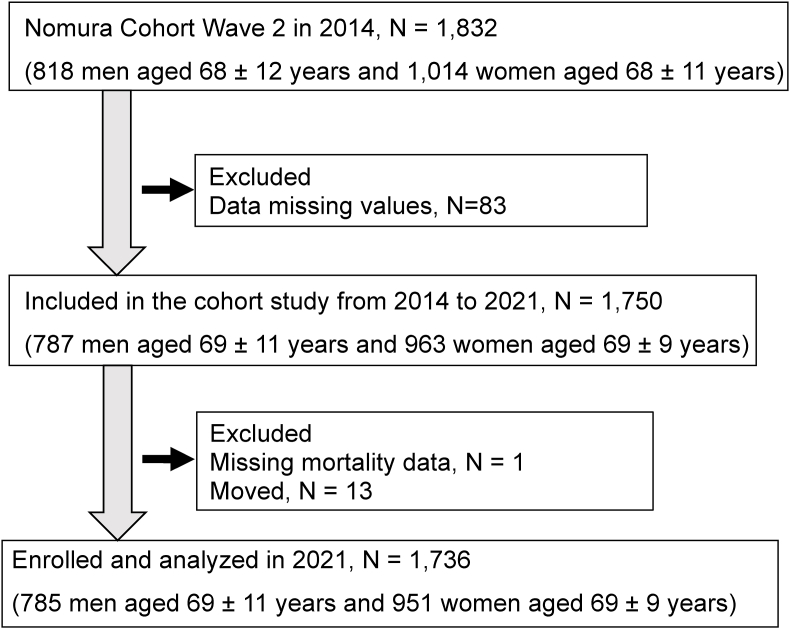
Nomura cohort.

This analysis was part of the Nomura study [7,24], which was conducted on individuals aged ≥20 years who had participated in a community-based annual checkup at the Nomura Health and Welfare Center in a rural town in Ehime Prefecture, Japan. The study used a structured questionnaire to collect data on the participants’ physical activity (e.g., exercise habits), medical history, current condition, and medication (e.g., antihypertensive, antidyslipidemic, antidiabetic, and UA-lowering medication). Plasma samples obtained following an overnight fast were available for all participants. Fig. 1 provides a flowchart of participant inclusion and exclusion. After the baseline examination, the 1736 were followed up for 7 years. Confirmation of whether the individual was living or had died was obtained by Basic Resident Register. This study was conducted in compliance with the Declaration of Helsinki and was approved by the ethics committee of Ehime University School of Medicine (Institutional Review Board [IRB]: no. 1903018). Written informed consent was obtained from all participants.

### 2.2 Evaluation of risk factors

Data on the participants' demographic characteristics and risk factors were obtained from clinical files. Their body mass index (BMI) was calculated as weight (kg) divided by the square of their height (m). Slenderness was defined as BMI <18.5 kg/m^2^. Smoking status was determined based on whether a participant was currently a smoker. Participants were classified as current drinkers if they had recently consumed at least one standard alcoholic beverage. Blood pressure was measured using a cuff bladder whose size was adapted to the circumference of the participant's arm. Systolic blood pressure (SBP) and diastolic blood pressure (DBP) were measured using an automatic blood pressure recorder attached to the participant's right upper arm. Prior to the measurement, the participants were asked to remain in a seated position and rest for at least 5 min. Those who had an SBP ≥140 mmHg, had a DBP ≥90 mmHg, or used antihypertensive medication were considered to have hypertension. Triglycerides (TG), high-density lipoprotein cholesterol (HDL-C), low-density lipoprotein cholesterol (LDL-C), fasting plasma glucose (FPG), creatinine (Cr), and SUA were measured during fasting. Serum uric acid was determined by the uricaseperoxidase method using Atellica CH analyzer (Siemens Healthineers, Japan). The participants were classified as having hypertriglyceridemia if their TG levels were ≥150 mg/dL, low HDL cholesterolemia if their HDL-C levels were <40 mg/dL, and high LDL cholesterolemia if their LDL-C levels were ≥140 mg/dL or if they were on antidyslipidemic medication. Participants whose hemoglobin A1c was 6.5% or higher or who were on antidiabetic medication were considered diabetic.

We divided the SUA levels into three categories. For male participants, these categories were “low” (<3.5 mg/dL), “medium” (3.5–7.9 mg/dL), and “high” (≥8.0 mg/dL). For female participants, the equivalent categories were <3.0 mg/dL, 3.0–6.9 mg/dL, and ≥7.0 mg/dL. Their estimated glomerular filtration rate (eGFR) was calculated using the Chronic Kidney Disease Epidemiology Collaboration (CKD-EPI) equations modified using a Japanese coefficient. Males: Cr ≤ 0.9 mg/dL, 141 × (Cr/0.9)^−0.411^ × 0.993^age^ × 0.813; Cr > 0.9 mg/dL, 141 × (Cr/0.9)^−1.209^ × 0.993^age^ × 0.813; females: Cr ≤ 0.7 mg/dL, 144 × (Cr/0.7)^−0.329^ × 0.993^age^ × 0.813; Cr > 0.7 mg/dL, 144 × (Cr/0.7)^−1.209^ × 0.993^age^ × 0.813 [25]. Proteinuria and/or an eGFR value of 60 mL/min/1.73 m^2^ or less were defined as chronic kidney disease (CKD). CVD included ischemic heart disease, ischemic stroke, and peripheral vascular disease.

### 2.3 HGS test

To measure their HGS, the participants were asked to hold a dynamometer in the hand that was to be examined, with the elbow bent at a right angle and held close to their body. The participants had to place the first metacarpal of all four fingers over the outer handle of the dynamometer and the middle metacarpal over the inner handle. They then had to grip the dynamometer with isometric maximal muscle strength and hold it for approximately 5 s. They were instructed to keep the rest of their body still. The average of the measurements for the two hands was used for analysis. Low HGS was defined as HGS <30.0 kg for male participants and HGS <20.0 kg for female participants [26].

### 2.4 Statistical analysis

All normally distributed continuous variables are expressed as mean ± standard deviation (SD), but we report median with interquartile range for non-normally distributed variables (e.g., TG, HbA1c, and HGS). In all the analyses, parameters with non-normal distributions were used after log-transformation. Differences in means and prevalence among the groups were analyzed by ANOVA for continuous data and χ^2^ test for categorical data, respectively. Pearson's correlations were calculated in order to identify the associations among various characteristics. We modeled the relationships between baseline characteristics and all-cause mortality using Cox proportional hazards regressions with age as the timescale. We controlled for possible confounding by the following factors: gender, age, BMI, smoking status, drinking status, history of CVD, hypertension, hypertriglyceridemia, low HDL-cholesterolemia, high LDL-cholesterolemia, diabetes, CKD, low HGS, and the three SUA categories.

The participants were divided into six groups according to HGS (low and high) and SUA (low, medium, and high). We modeled the relationships between these groups and all-cause mortality using Cox proportional hazards regressions with age as the timescale, and the group that had the lowest hazard ratio (HR) was used for all-cause mortality as the reference group. In addition, a sensitivity analysis stratified by gender, presence or absence of exercise, presence or absence of CKD, presence or absence of UA-lowering medication, and time to mortality was performed to confirm the suspected association between the groups and all-cause mortality. To test for the possible effect of reverse causation, we also carried out an analysis where was excluded participants who passed away within the first two years of the follow-up period. All *p*-values were two-tailed, and *p* < 0.05 was considered significant. IBM SPSS Statistics version 27 (SPSS, Chicago, IL, United States) was used for all statistical analyses.

## 3 Results

### 3.1 Baseline characteristics of participants by SUA levels

Table 1 presents the participants’ baseline characteristics according to the SUA levels. In total, we included 785 men aged 69 ± 11 years (range: 24–90) and 951 women aged 69 ± 9 years (range: 26–90) in the study. The results indicate that male gender, slenderness, current drinker status, hypertension, hypertriglyceridemia, high LDL-cholesterolemia, and CKD were associated with SUA levels. We found no association between age and SUA levels.

**Table 1:** Baseline characteristics of participants by SUA levels.

**Men N** = **785**	Low SUA	Medium SUA	High SUA	***p***
	**< 3.5**	**≥ 3.5 < 8.0**	**≥ 8.0 mg/dL**	
**Women N** = **951**	**< 3.0**	**≥ 3.0 & < 7.0**	**≥ 7.0 mg/dL**	
**Baseline characteristics N** = **1736**	**N** = **64**	**N** = **1593**	**N** = **79**	
Male gender	24 (37.5)	709 (44.5)	52 (65.8)	<0.001
Age (years)	69 ± 10	69 ± 11	69 ± 9	0.231
Slenderness, n (%)	9 (14.1)	108 (6.8)	1 (1.3)	**0.010**
Body mass index (kg/m^2^)	21.5 ± 2.8	22.8 ± 3.1	24.5 ± 3.5	**< 0.001**
Current smoker, n (%)	6 (9.4)	146 (9.2)	11 (13.9)	0.367
Current drinker, n (%)	15 (23.4)	423 (26.6)	39 (49.4)	**< 0.001**
Exercise status, n (%)	29 (45.3)	582 (36.5)	31 (39.2)	0.330
History of cardiovascular disease, n (%)	4 (6.3)	105 (6.6)	6 (7.6)	0.934
Hypertension, n (%)	44 (68.8)	1002 (62.9)	61 (77.2)	**0.025**
Systolic blood pressure (mmHg)	138 ± 19	135 ± 18	138 ± 15	0.221
Diastolic blood pressure (mmHg)	78 ± 11	78 ± 10	80 ± 11	0.114
Antihypertensive medication, n (%)	26 (40.6)	686 (43.1)	46 (58.2)	**0.026**
Hypertriglyceridemia, n (%)	9 (14.1)	222 (13.9)	21 (26.6)	**0.006**
Triglycerides (mg/dL)	77 (60–120)	88 (67–122)	96 (70–152)	**0.006**
Low HDL-cholesterolemia, n (%)	9 (14.1)	153 (9.6)	12 (15.2)	0.149
HDL cholesterol (mg/dL)	66 ± 17	66 ± 17	61 ± 16	**0.044**
High LDL-cholesterolemia, n (%)	20 (31.3)	711 (44.6)	25 (31.6)	**0.010**
LDL cholesterol (mg/dL)	117 ± 32	120 ± 29	108 ± 32	**0.002**
Lipid-lowering medication, n (%)	8 (12.5)	358 (22.6)	13 (16.5)	0.083
Diabetes, n (%)	7 (10.9)	190 (11.9)	14 (17.7)	0.292
Hemoglobin A1c (%)	5.7 (5.4–5.8)	5.7 (5.4–5.9)	5.7 (5.4–6.1)	0.789
Antidiabetic medication, n (%)	5 (7.8)	130 (8.2)	12 (15.2)	0.089
Chronic kidney disease, n (%)	9 (14.1)	309 (19.4)	38 (48.1)	**< 0.001**
eGFR (mL/min/1.73 m^2^)	76.6 ± 7.8	72.2 ± 11.5	61.8 ± 18.9	**< 0.001**
Proteinuria, n (%)	8 (12.5)	158 (9.9)	17 (21.5)	**0.004**
SUA (mg/dL)	2.5 ± 0.6	5.3 ± 1.1	8.3 ± 0.9	**< 0.001**
UA lowering medication (%)	0	64 (4.0)	4 (5.1)	0.231
Low handgrip strength, n (%)	24 (37.5)	514 (32.3)	28 (35.4)	0.585
Handgrip strength (kg)	25.6 ± 8.4	27.0 ± 8.6	29.8 ± 10.0	**0.010**

SUA, serum uric acid; HDL, high-density lipoprotein; LDL, low-density lipoprotein; eGFR, estimated glomerular filtration ratio. Data presented are means ± standard deviation. Data for triglycerides, hemoglobin A1c, and handgrip strength were skewed, are presented as median (interquartile range) values, and were log-transformed for analysis. *P-values are from ANOVAs for continuous variables or χ^2^ tests for categorical variables.

### 3.2 Relationship between baseline HGS and SUA levels by gender

**Fig. 2:**
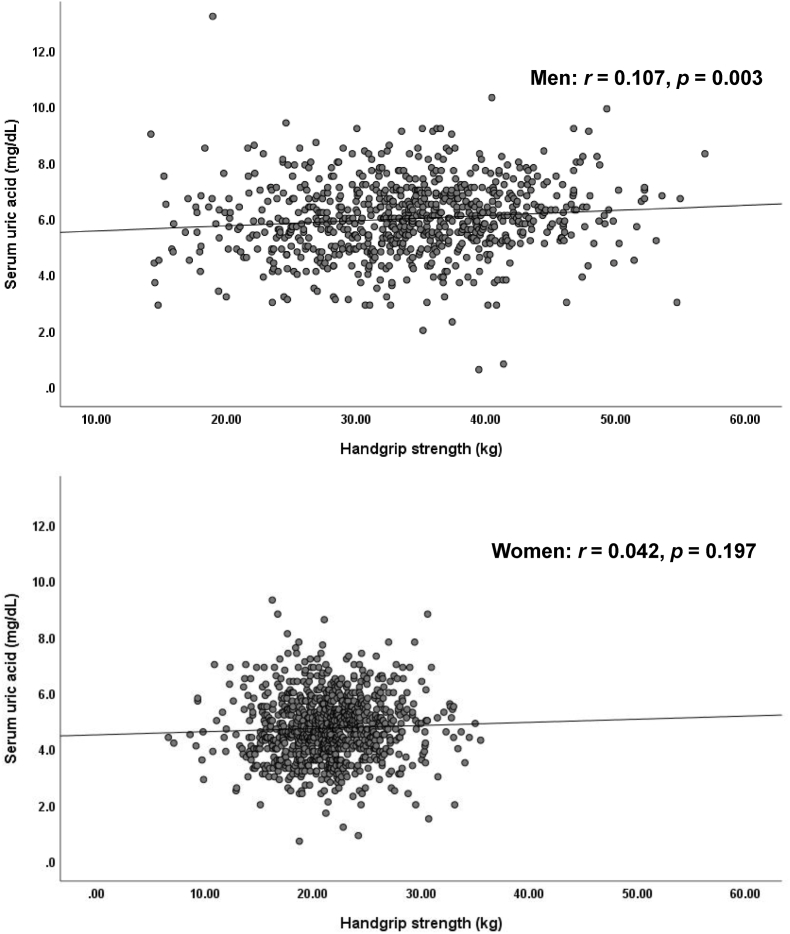
Relationship between baseline HGS and SUA by gender There was a significant correlation between HGS and SUA levels in the male participants (*r* = 0.107, *p* = 0.003), but not in the female participants (*r* = 0.042, *p* = 0.197).

Fig. 2 illustrates the relationship between HGS and SUA. There was a significant positive relationship between HGS and SUA among the male participants (r = 0.017, *p* = 0.003). but not in the female participants (*r* = 0.042, *p* = 0.197).

### 3.3 The heat map of Pearson's correlation coefficients among baseline characteristics

**Fig. 3:**
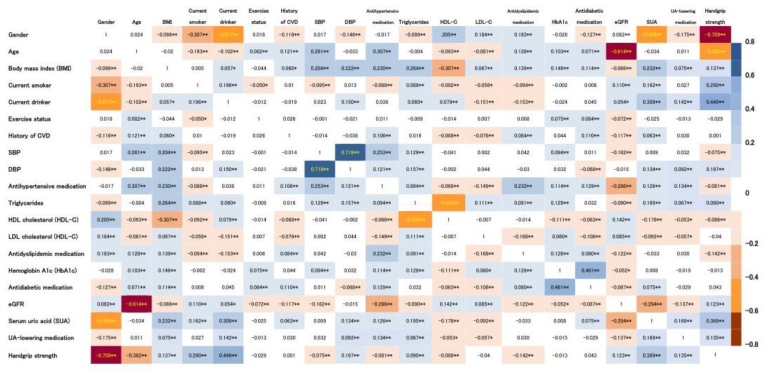
The heat map of Pearson's correlation coefficients among baseline characteristics CVD, cardiovascular disease. Data for triglycerides, hemoglobin A1c, and handgrip strength were skewed and were log-transformed for analysis. *P*-values are from Pearson's correlation coefficient. *P < 0.05, **P < 0.01. Fields in blue and red indicate positive and negative correlations between the characteristics. Numbers in the fields represent Pearson's correlation coefficients (r). (For interpretation of the references to colour in this figure legend, the reader is referred to the Web version of this article.)

As shown in Fig. 3, SUA was positively associated with BMI, current smoker, current drinker, history of CVD, DBP, antihypertensive medication, TG, antidiabetic medication, and UA-lowering medication, and was negatively associated with gender, HDL-C, LDL-C, and eGFR.

### 3.4 Association of baseline characteristics with all-cause mortality

Table 2 lists the HRs and 95% confidence intervals (CI) for the associations between the baseline characteristics and all-cause mortality in the univariable and multivariable analyses. The former revealed positive associations between all-cause mortality and the low (HR: 2.03; 95% CI: 1.03–4.00) and high (HR: 2.05; 95% CI: 1.11–3.81) SUA categories, low HGS (HR: 3.91; 95% CI: 2.76–5.55), and male gender, aging, slenderness, lack of exercise, and CKD. The multivariable analysis revealed a significant and positive association between all-cause mortality and low HGS (HR: 1.96; 95% CI: 1.31–2.92), the low SUA category (HR: 2.13; 95% CI: 1.06–4.28), and other confounders.

**Table 2:** Relationship between baseline characteristics and all-cause mortality.

Baseline characteristics N = 1736	Univariable		Multivariable	
	HR (95% CI)	*p*	HR (95% CI)	*p*
Gender (male = 1, women = 2)	**0.52 (0.37**–**0.73)**	**< 0.001**	**0.50 (0.33**–**0.76)**	**0.001**
Age (per 1 year increment)	**1.12 (1.10**–**1.15)**	**< 0.001**	**1.09 (1.06**–**1.12)**	**< 0.001**
Slenderness (no = 0, yes = 1)	**2.08 (1.25**–**3.47)**	**0.005**	**2.59 (1.51**–**4.43)**	**0.001**
Current Smoker (no = 0, yes = 1)	0.67 (0.34–1.33)	0.253	0.98 (0.48–2.00)	0.954
Current drinker (no = 0, yes = 1)	1.14 (0.79–1.64)	0.499	1.04 (0.68–1.59)	0.868
Exercise status (no = 0, yes = 1)	**0.65 (0.45**–**0.95)**	**0.024**	**0.56 (0.38**–**0.81)**	**0.002**
History of cardiovascular disease (no = 0, yes = 1)	**1.95 (1.16**–**3.28)**	**0.012**	1.11 (0.65–1.90)	0.712
Hypertension (no = 0, yes = 1)	**1.88 (1.27**–**2.80)**	**0.002**	1.24 (0.82–1.82)	0.312
Hypertriglyceridemia (no = 0, yes = 1)	1.08 (0.68–1.72)	0.752	1.19 (0.71–1.98)	0.508
Low HDL-cholesterolemia (no = 0, yes = 1)	0.87 (0.48–1.57)	0.642	0.68 (0.36–1.27)	0.224
High LDL-cholesterolemia (no = 0, yes = 1)	0.85 (0.60–1.20)	0.352	1.26 (0.86–1.84)	0.230
Diabetes (no = 0, yes = 1)	1.34 (0.84–2.14)	0.215	1.13 (0.69–1.83)	0.637
Chronic kidney disease (no = 0, yes = 1)	**2.77 (1.97**–**3.90)**	**< 0.001**	**1.51 (1.04**–**2.20)**	**0.030**
SUA categories (medium = 0, low = 1)	**2.03 (1.03**–**4.00)**	**0.041**	**2.13 (1.06**–**4.28)**	**0.033**
SUA categories (medium = 0, high = 1)	**2.05 (1.11**–**3.81)**	**0.023**	1.74 (0.91–3.37)	0.095
Low handgrip strength (no = 0, yes = 1)	**3.91 (2.76**–**5.55)**	**< 0.001**	**1.96 (1.31**–**2.92)**	**0.001**

HR, hazard ratio; CI, confidence interval. Significant values (*p* < 0.05) are presented in bold.

### 3.5 Kaplan–Meier survival curves for the association of baseline SUA levels with all-cause mortality according to HGS

**Fig. 4:**
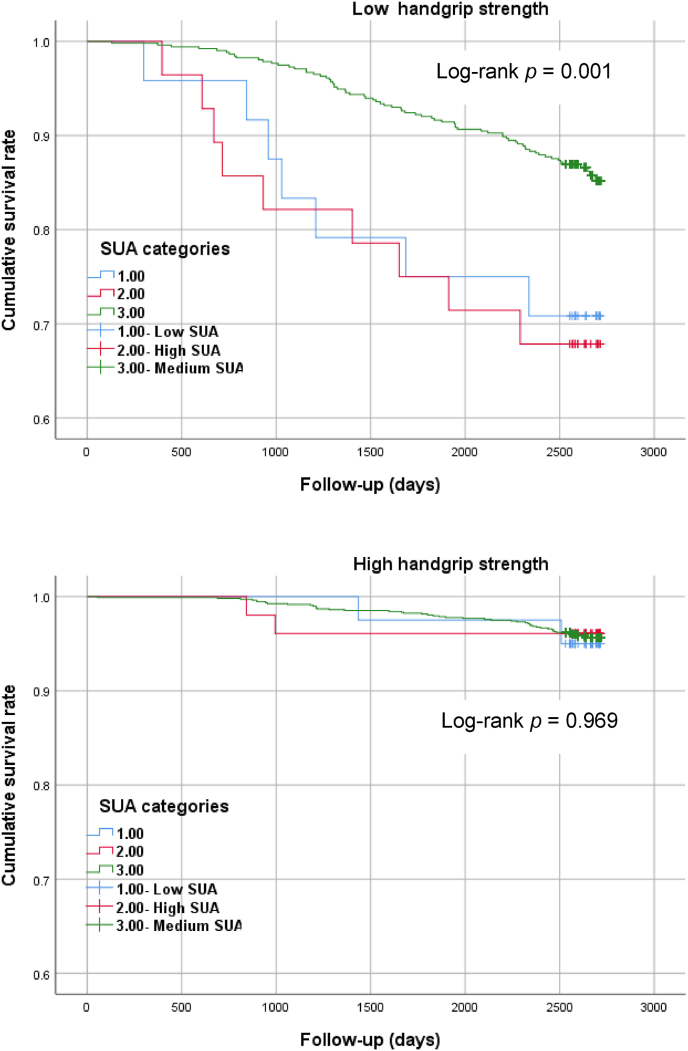
Kaplan–Meyer survival analysis of the association between baseline SUA levels and all-cause mortality, stratified according to HGS, over a follow-up period of over seven years. *P-*values are based on using a log-rank test of equality across categories.

The Kaplan–Meier survival curves in Fig. 4 reveal **patterns in the association of the SUA levels with all-cause** mortality according to HGS. Among participants with low HGS (<30.0 kg in males, <20.0 kg in females), the cumulative survival rate was significantly lower in those with low (<3.5 mg/dL in males, <3.0 in females) and high SUA levels (≥8.0 mg/dL in males, ≥7.0 mg/dL in females) than in the reference group (3.5–7.9 mg/dL in males, 3.0–6.9 mg/dL in females; log-rank test *p* < 0.001). Among participants with high HGS (≥30.0 kg in males, ≥20.0 kg in females), there was no difference in the cumulative survival rates of the three SUA-category groups.

### 3.6 Combined effect of HGS and SUA levels on risk of all-cause mortality

**Fig. 5:**
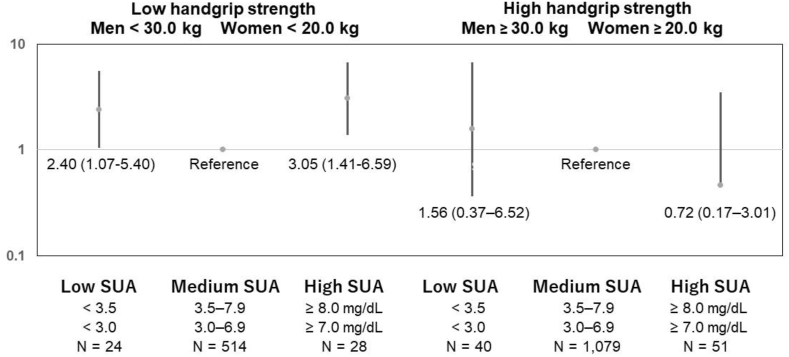
Association between baseline SUA levels and all-cause mortality based on HGS.

As shown in Fig. 5, among participants with low HGS, those with low (HR: 2.40; 95% CI: 1.07–5.40) and high SUA levels (HR: 3.05; 95% CI; 1.41–6.59) had a significantly higher HR for all-cause mortality than the reference group. Among participants with high HGS, there were no significant differences in the HR between the three SUA-category groups. Table 3 reveals a significant interaction effect between SUA levels and HGS on all-cause mortality risk. For the entire sample, compared with participants with high HGS and medium SUA levels (reference), all three SUA-category groups of participants with low HGS had a higher HR (95% CI) for all-cause mortality (4.30 [1.85–10.0]; 1.78 [1.17–2.71]; 4.24 [1.93–9.32]).

**Table 3:** Combined effect of baseline HGS and SUA levels on risk of all-cause mortality.

**Men**	Low handgrip strength Men <30.0 kg Women <20.0 kg			High handgrip strength Men ≥30.0 kg Women ≥20.0 kg		
	Low SUA	Medium SUA	High SUA	Low SUA	Medium SUA	High SUA
	**< 3.5**	**3.5**–**7.9**	**≥ 8.0 mg/dL**	**< 3.5**	**3.5**–**7.9**	**≥ 8.0 mg/dL**
**Women**	**< 3.0**	**3.0**–**6.9**	**≥ 7.0 mg/dL**	**< 3.0**	**3.0**–**6.9**	**≥ 7.0 mg/dL**
**N** = **1736**	**N** = **24**	**N** = **514**	**N** = **28**	**N** = **40**	**N** = **1079**	**N** = **51**
Low + high handgrip strength						
HR (95% CI)	**4.30 (1.85**–**10.0)**	**1.78 (1.17**–**2.71)**	**4.24 (1.93**–**9.32)**	1.56 (0.38–6.48)	Reference	0.83 (0.20–3.44)
*P*	**0.001**	**0.008**	**< 0.001**	0.537	–	0.794

SUA, serum uric acid; HR, hazard ratio; CI, confidence interval. Multivariate-adjusted for confounding factors as per Table 2. Significant values (*p* < 0.05) are presented in bold.

### 3.7 Combined effect of HGS and SUA levels on risk of all-cause mortality in sub-analyses

Table 4 stratifies participants based on gender, exercise, CKD, UA lowering medication, and time to death (<730 days, ≥730 days). Our analysis confirmed that SUA levels among participants with low HGS were significantly associated with all-cause mortality in the group with survival of ≥730 days. This association was significant in both genders, regardless of the effect of CKD, and in the group without UA-lowering medication. This analysis also revealed that the combination of low HGS and SUA level is associated with all-cause mortality.

**Table 4:** Combined effect of baseline HGS and SUA levels on the risk of all-cause mortality by sub-analysis.

	Low handgrip strength Men <30.0 kg Women <20.0 kg			High handgrip strength Men ≥30.0 kg Women ≥20.0 kg			
	**Low SUA**	**Medium SUA**	High SUA	**Low SUA**	**Medium SUA**	**High SUA**	
**Men**	**< 3.5**	**3.5**–**7.9**	**≥ 8.0 mg/dL**	**< 3.5**	**3.5**–**7.9**	**≥ 8.0 mg/dL**	***P* for**
**Women**	**< 3.0**	**3.0**–**6.9**	**≥ 7.0 mg/dL**	**< 3.0**	**3.0**–**6.9**	**≥ 7.0 mg/dL**	**interaction**
Gender							
Men (*N* = 785)	**4.58 (1.50**–**14.0)**	1.43 (0.83–2.48)	**4.05 (1.56**–**10.5)**	**------**	Reference	0.51 (0.07–3.79)	0.633
Women (*N* = 951)	**4.87 (1.26**–**18.9)**	**2.36 (1.20**–**4.67)**	**4.87 (1.02**–**23.2)**	3.07 (0.69–13.7)	Reference	2.77 (0.35–21.7)	
Exercise							
No (*N* = 1094)	**4.79 (1.73**–**13.2)**	**2.15 (1.31**–**3.54)**	**6.50 (2.52**–**16.8)**	1.36 (0.18–9.99)	Reference	1.16 (0.27–4.89)	0.658
Yes (*N* = 642)	1.93 (0.37–9.99)	1.06 (0.47–2.39)	2.21 (0.52–9.43)	2.04 (0.26–16.1)	Reference	–	
Chronic kidney disease							
No (*N* = 1380)	2.55 (0.74–8.81)	**2.19 (1.29**–**3.71)**	**14.0 (4.12**–**47.5)**	1.03 (0.14–7.59)	Reference	1.07 (0.14–8.00)	0.923
Yes (*N* = 356)	**26.7 (5.92**–**120)**	1.11 (0.55–2.23)	1.90 (0.67–5.39)	5.56 (0.69–44.9)	Reference	0.48 (0.06–3.75)	
UA lowering medication							
No (*N* = 1668)	**4.53 (1.94–10.6)**	**1.92 (1.24–2.96)**	**4.33 (1.88–9.95)**	1.62 (0.39–6.72)	Reference	0.91 (0.22–3.80)	–
Yes (*N* = 68)	–	–	–	–	–	–	
Time to death							
<730 days (*N* = 34)	–	–	–	–	–	–	–
≥730 days (*N* = 1702)	**4.24 (1.72**–**10.4)**	**1.71 (1.11**–**2.64)**	2.27 (0.84–6.13)	1.67 (0.40–6.93)	Reference	0.84 (0.20–3.49)	

Multivariable adjusted for confounding factors as per Table 2. Significant values (*p* < 0.05) are presented in bold.

## 4 Discussion

This prospective cohort study showed that, after potential confounders have been adjusted for, HGS and SUA levels were associated with all-cause mortality in Japanese male and female adults with varying HGS. Further, SUA levels at both ends of the spectrum were associated with an increased risk of all-cause mortality in participants with low HGS. However, among participants with high HGS, none of the SUA level categories was associated with a significant risk of all-cause mortality. To the best of our knowledge, the effect of the interaction between HGS and SUA on all-cause mortality has rarely been quantified in previous epidemiology studies on community-dwelling people.

The association between hyperuricemia and various health outcomes has been highlighted in numerous prospective and cross-sectional studies [[1], [2], [3], [4], [5], [6], [7]]. However, SUA levels are also strongly correlated with gender, age, obesity, insulin resistance, hypertension, diabetes, metabolic abnormalities, and renal function, and the causal role of SUA in all-cause mortality remains controversial. There are conflicting findings concerning the association between SUA and all-cause mortality, particularly whether high or low SUA levels negatively impact health. While some studies have reported that there is a link between SUA and all-cause mortality only in men [27], others have observed this association only in women [28] or in both genders [29,30]. Further, some research has indicated that the relationship is U-shaped for men but J-shaped for women [31], while other findings suggest that it is J-shaped for both [30]. In the present study, we recorded mean ± SD SUA levels of 6.0 ± 1.3 mg/dL for male participants and 4.7 ± 1.1 mg/dL for female participants, and based on these means ± 2 SD we classified the participants into three groups. Our results revealed a U-shaped relationship between SUA levels and all-cause mortality among both male and female participants with low HGS.

Our results, particularly is the finding of a significant association between low 10.13039/100004315HGS and increased risk of all-cause mortality, are supported by previous studies. A 10-low year prospective cohort study on 9229 middle-aged and older Korean individuals (of whom 4131 were male and 5098 female) showed that there was a strong association between muscular weakness measured via HGS and a higher risk of all-cause mortality. This association was specifically observed in the lowest quartile (HR: 2.34, 95% CI: 1.80–3.05) and in the muscle weakness group (HR: 1.80, 95% CI: 1.52–2.13) in the fully adjusted model [32]. A meta-analysis of 30 studies involving 194,767 older adult participants indicated that a greater HGS was associated with an 18% reduction in all-cause mortality, and among older women, the lowest HGS that was not associated with an increased risk of all-cause mortality was 18.21 kg [33]. Handgrip strength can therefore be considered an accurate and consistent predictor of all-cause mortality in middle-aged and older adults. Our study also demonstrated that compared with participants with high HGS, those with low HGS had a significantly higher risk of mortality.

One study identified cutoff values for weak HGS in Japan of 30.3 kg for men and 19.3 kg for women, and people with HGS below these cutoffs represented fewer than 25% of the participants of that study [34]. In line with the European Working Group on Sarcopenia in Older People, Kim et al. [35] proposed cutoff values for weak HGS of 28.9 kg for men and 16.8 kg for women. Similarly, Yoo et al. [36] determined cutoff values for weak HGS of 28.6 kg and 16.4 kg for healthy elderly Korean men and women, respectively. In this study, we defined low HGS as < 30.0 kg for men and <20.0 kg for women [26,34,37].

This study confirmed that the interaction between HGS and SUA levels has a significant effect on the risk of all-cause mortality after adjusting for multiple potential confounders and accounting for the possibility of reverse causation. Further, we found a nonmonotonic association between SUA levels and mortality in participants with low HGS. This suggests that HGS modifies the association between SUA levels and mortality risk. It is possible that processes underlying muscle weakness are involved in the pathway linking high and low SUA levels to health status. In addition, muscle weakness may be involved in pathways linking high and low SUA levels and health risks, driving the specificity of the relationship between the two [38].

There are several possible explanations for the association between low HGS and all-cause mortality risk [21]. First, low SUA levels are associated with low HGS and this contributes to an additive increase in all-cause mortality risk [39]. Second, low HGS is significantly associated with increased incidence of chronic diseases, thereby additively contributing to an increased risk of all-cause mortality. Third, low HGS may reflect frailty because it is an important component of the phenotype. Frail older adults are less likely to have access to medication and treatment when needed [40], which further contributes to the increased risk of early mortality from all causes. Skeletal muscle is an endocrine organ and when it weakens, the release of several cytokines and peptides (i.e., myokines) into the blood is reduced. This is associated with increased inflammation [41], which contributes to death.

### 4.1 Strength and limitation of the study

A key strength of this study is its accuracy due to its character as a long-term follow-up study with a fixed number of participants and our adjustment for several potential confounding factors. However, the study did have some limitations. First, it was an observational study, and the sample was limited to generally healthy adults aged on average 69 (±10) years who had undergone a health examination. Further, the participants lived in rural areas of Japan, where the population is aging rapidly. This sample therefore did not well represent the overall population. Second, the study took account only of deaths that were recorded in the Basic Resident Registry, which would not have occurred for people who had relocated away from the area during the period in question. Third, the potential influence of medications such as antihypertensive, antidyslipidemic, antidiabetic, and SUA-lowering medications, underlying diseases, metabolic syndrome, and changes in lifestyle at baseline and during follow-up may have biased our results. Fourth, renal function was assessed based on eGFR and proteinuria, not on urinary albumin data. Finally, the strength of the combined effect of SUA levels and HGS on mortality may have been underestimated because of the relatively small sample size.

## 5 Conclusions

This study revealed a nonlinear (U-shaped) association between serum uric acid and all-cause mortality risk. This relationship varied with HGS: the lower the HGS, the stronger the association. Our findings suggest that in a clinical setting, simply measuring HGS may help to identify adults with either high or low SUA levels and who are at a particularly high risk of experiencing adverse health events. Additional prospective studies involving healthy community-dwelling people from the general population are necessary to further examine the mechanisms behind this association and to determine if interventions like lifestyle modifications and SUA-controlling medications for adults would improve health outcomes.

## Ethics approval

The study was approved by the Ethics Committee of the Ehime University Graduate School of Medicine (IRB: no. 1903018). Informed consent was obtained from all participating subjects in the study.

## Funding

This work was partially supported by a Grant-in-Aid for Scientific Research from the Foundation for Development of Community (2022). No additional external funding was received. The funders played no role in the study design, data collection and analysis, decision to publish, or manuscript preparation.

## CRediT authorship contribution statement

**Ryuichi Kawamoto:** Formal analysis, Writing – original draft, Funding acquisition, Conceptualization, Writing – original draft. **Asuka Kikuchi:** Formal analysis. **Daisuke Ninomiya:** Data curation. **Teru Kumagi:** Supervision.

## Declaration of competing interest

The authors declare that they have no competing interests.

## List of abbreviations

UA: uric acid
CVD: cardiovascular diseases
SUA: serum uric acid
HGS: handgrip strength
IRB: Institutional Review Board
BMI: body mass index
SBP: systolic blood pressure
DBP: diastolic blood pressure
TG: triglyceride
HDL-C: high-density lipoprotein cholesterol
LDL-C: low-density lipoprotein cholesterol
FPG: fasting plasma glucose
Cr: creatinine
eGFR: estimate the glomerular filtration ratio
CKD-EPI: Chronic Kidney Disease Epidemiology Collaboration
CKD: Chronic Kidney Disease
SD: standard deviation
HR: hazard ratio
CI: confidence interval
